# Reply to: The pitfalls of interpreting hyperintense FLAIR signal as lymph outside the human brain

**DOI:** 10.1038/s41467-023-40510-8

**Published:** 2023-08-16

**Authors:** Mehmet Sait Albayram, Garrett Smith, Onder Albayram

**Affiliations:** 1 Department of Radiology, University of Florida College of Medicine, Gainesville, FL 32610 USA; 2 Department of Pathology and Laboratory Medicine, Medical University of South Carolina, Charleston, SC 29425 USA; 3 Department of Neuroscience, Medical University of South Carolina, Charleston, SC 29425 USA

**Keywords:** Neuroscience, Medical imaging

**replying to** G. Ringstad & P. K. Eide *Nature Communications* 10.1038/s41467-023-40508-2 (2023)

We thank Dr. Ringstad and his colleague for their interest in our article^1^. We are happy to address their concerns^2^.

Mezey et al.^3^ showed that lymphatic marker-positive cells (LMPCs) were present in the walls of arteries and veins, the pia mater, the arachnoid, the venous sinuses, and among the layers of the dura mater. Many LMPCs were also present in the perineurium and endoneurium of cranial nerves. Furthermore, Aspelund et al.^4^ showed a rich lymphatic network in the dura along the middle meningeal artery, anterior cranial fossa, and nasal cavity (Aspelund et al., 2015; Fig. 1I, J). Some lymphatic structures were comparable in size to the middle meningeal artery (Aspelund et al., 2015; Fig. 1C). Therefore, according to our data and recent histological evidence support, we hypothesize that lymphatic structures or lymph fluid are more abundant than what Ringstad et al. proposed in their letter^1^ and parasagittal dura paper^5^ (Ringstad et al., 2020; Figure 6).

We do not directly claim to see lymphatic vessels with MR but rather waste/macromolecular-rich fluid densities in the lymphatic structures. In addition, a dynamic 3D T1 IV gadolinium MR study was published on July 1, 2022, in JEM by Jacob et al.^6^ 6 months later than our paper. They used the T1 SPACE DANTE MR sequence to accurately segregate the slow-flow circuits of the lymphatic vessels from the faster-flow circuits of arteries, veins, and venules. These techniques present minimal artifact and contrast-based signal detection. Their findings corroborate our hypothesized dorsal lymphatic flow and structures and further suggest significant ventral lymphatic flow at the skull base in humans at the level of jugular foremen, sellar region, and 5th nerve pathway. Although we detected strong signal in the anterior cranial fossa around the olfactory nerves and at the level of the internal auditory canal, Jacob et al. did not detect any lymphatic signal at these regions. These differences might relate to internal marker variances (gadolinium vs. solid-rich interstitial fluid). However, we cannot completely rule out possible artifacts in these regions. Therefore, we agree that prospective and well-designed live-human studies supported by histopathology are needed for clarification. They did not report on the internal auditory canal region-CPA cistern. Given the available evidence, we conclude that the dural FLAIR signal at the level of the internal auditory canal is likely a lymphatic signal similar to other dural surfaces. However, our signal extends beyond the dura into subdural or subarachnoid spaces and may represent artifacts or CSF-ISF exchange in this region toward the dural lymphatics system. More definitive data will be required in future studies to confirm the presence of CSF-ISF exchange in this region. Recently, subarachnoid lymphatic structures have been shown by Mezey et al. 2021^3^ but more investigation is needed to confirm this finding. The possibility of the artifact at the level of the internal auditory canal in the subdural and subarachnoid spaces could not be excluded. Further histopathological studies will be needed.

Ringstad et al. additionally commented that linear hyperintense FLAIR signal in the suprasellar cistern could not be seen in corresponding T1 weighted images. Therefore, they concluded that the linear signal within the CSF does not represent dura^2^. We dispute this claim as the optic-chiasmatic groove dura, and diaphragma sella are located precisely in this region. Our FLAIR signal follows the chiasmatic groove/diaphragma sella as shown in our paper Fig. 4c^1^ (diaphragma sella indicated by a double arrow) (Fig. 1A). The diaphragma sella can only be seen well on T1 weighted series with contrast. However, we identify a subtle linear pattern, most likely the diaphragma sella (yellow arrowhead) in Ringstad et al. Figure 1c^2^, just under their arrow (with contrast adjustment) (Fig. 1B). In addition, Jacob et al.^6^ reported that meningeal lymphatic vessels were identified along the inter cavernous regions-sellar region in animals. Ringstad et al. also question our proposal that the smooth continuous signal along the wall of the jugular vein represents a lymphatic-type signal^2^. Our paper references autopsy findings of lymphatic vessels connecting the lymph nodes adjacent to the internal jugular vein within the carotid sheath^7^, which is consistent with our observed patterns of continuous signal from the superior sagittal sinus to the jugular vein and the lymph nodes in the neck (page 9, left column, paragraph two, line 4). We observed peri-arterial signal in our study, but these data were abridged from earlier manuscript drafts per reviewer suggestion. We deduce that our signals in the jugular veins do not represent flow within smaller veins. If this signal were related to veins, we would expect a more prominent signal, as veins are larger and more central. Our observed signals, however, are more prominent at the skull base, show continuity with retropharyngeal and deep cervical lymph nodes, and sharply attenuate moving inferior to these structures. In addition, our observed signal shows a closer correspondence with lymph nodes and the reticular connections with lymph nodes, which we propose are likely afferent lymph vessels. The study by Jacob et al.^6^ also supported our findings, describing continuous lymphatic-slow flow contrast signal along the venous sinuses and jugular veins connected with cervical lymph nodes.

**Fig. 1:**
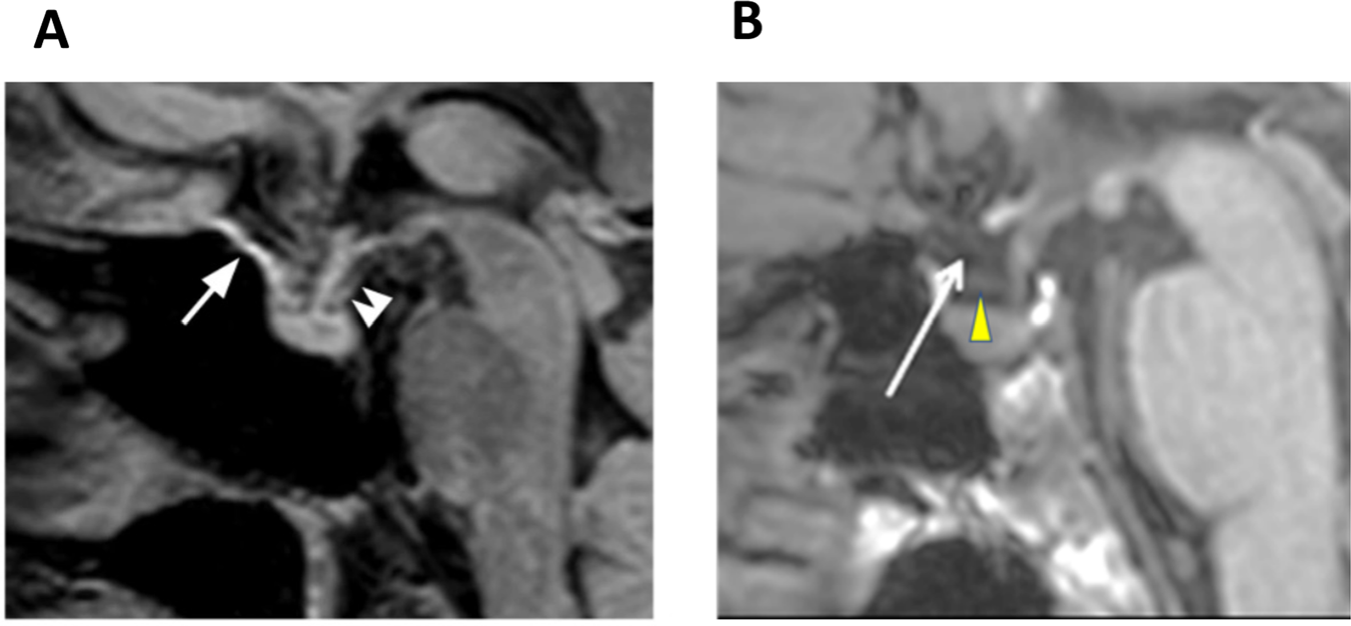
Visualization of the diaphragma sella. **A** Sagittal FLAIR images show the putative ventral dural lymphatic elements in the diaphragma sella (double arrowhead; (adapted from Figure 4c of Albayram et al., *Nat. Commun*. 13, 203 (2022)^1^). **B** Moreover, we can identify a subtle linear pattern, most likely the diaphragma sella (yellow arrowhead) in Ringstad et al. Figure 1c^2^, just under their arrow, as presented below (with contrast adjustment); (adapted from Fig. 1c at Rinstad et al., *Nat. Commun*. submitted).

We have also created the step-by-step protocols used in the original publication to the *Protocol Exchange*^8^.

We thank Dr. Ringstad et al.^2^ for their interest in our article. We hope our responses address their concerns.

## Reporting summary

Further information on research design is available in the Nature Portfolio Reporting Summary linked to this article.

### Supplementary information

Reporting Summary
